# Acceptability, Appropriateness, and Feasibility of Automated Screening Approaches and Family Communication Methods for Identification of Familial Hypercholesterolemia: Stakeholder Engagement Results from the IMPACT-FH Study

**DOI:** 10.3390/jpm11060587

**Published:** 2021-06-21

**Authors:** Laney K. Jones, Nicole Walters, Andrew Brangan, Catherine D. Ahmed, Michael Gatusky, Gemme Campbell-Salome, Ilene G. Ladd, Amanda Sheldon, Samuel S. Gidding, Mary P. McGowan, Alanna K. Rahm, Amy C. Sturm

**Affiliations:** 1 Genomic Medicine Institute, Geisinger, Danville, PA 17822, USA; nlwalters1@geisinger.edu (N.W.); ambrangan@geisinger.edu (A.B.); MGatusky@som.geisinger.edu (M.G.); gemme.campbell@ufl.edu (G.C.-S.); igladd@geisinger.edu (I.G.L.); ssgidding@geisinger.edu (S.S.G.); akrahm@geisinger.edu (A.K.R.); asturm@geisinger.edu (A.C.S.); 2 The FH Foundation, Pasadena, CA 91101, USA; cda@thefhfoundation.org (C.D.A.); ams@thefhfoundation.org (A.S.); mpm@thefhfoundation.org (M.P.M.); 3 Department of Advertising, University of Florida, Gainesville, FL 33620, USA; 4 Dartmouth Hitchcock Medical Center, Lebanon, NH 03756, USA

**Keywords:** familial hypercholesterolemia, identification, implementation outcomes, cascade screening, cascade testing, chatbots, direct contact

## Abstract

Guided by the Conceptual Model of Implementation Research, we explored the acceptability, appropriateness, and feasibility of: (1) automated screening approaches utilizing existing health data to identify those who require subsequent diagnostic evaluation for familial hypercholesterolemia (FH) and (2) family communication methods including chatbots and direct contact to communicate information about inherited risk for FH. Focus groups were conducted with 22 individuals with FH (2 groups) and 20 clinicians (3 groups). These were recorded, transcribed, and analyzed using deductive (coded to implementation outcomes) and inductive (themes based on focus group discussions) methods. All stakeholders described these initiatives as: (1) acceptable and appropriate to identify individuals with FH and communicate risk with at-risk relatives; and (2) feasible to implement in current practice. Stakeholders cited current initiatives, outside of FH (e.g., pneumonia protocols, colon cancer and breast cancer screenings), that gave them confidence for successful implementation. Stakeholders described perceived obstacles, such as nonfamiliarity with FH, that could hinder implementation and potential solutions to improve systematic uptake of these initiatives. Automated health data screening, chatbots, and direct contact approaches may be useful for patients and clinicians to improve FH diagnosis and cascade screening.

## 1. Introduction

Familial hypercholesterolemia (FH) is a common inherited cholesterol disorder that increases the risk for premature cardiovascular disease by causing lifelong exposure to high cholesterol [1,2,3]. Diagnostic criteria for FH have been established and effective treatments are available. Diagnosis can be made clinically, based on high cholesterol levels, family history, and physical findings, and by genetic testing [1,2]. However, in the U.S., only approximately 10% of individuals living with FH have been identified [4]. There are a variety of reasons for underdiagnosis of FH, including lack of systematic cholesterol screening in children and adults and a lack of awareness about FH among healthcare clinicians [5,6,7,8]. Two distinct and complementary initiatives in the U.S. to improve identification of FH include automated screening approaches of health-related data to identify those with possible FH and enhanced family communication methods and tools to improve cascade testing uptake by at-risk relatives [9,10,11,12,13,14]. 

Automated screening approaches have been used to predict and identify individuals who require screening for various health conditions [3,15,16,17]. Phenotype-based approaches use natural language processing and machine learning algorithms that utilize data from electronic health records (EHR), and health insurance claims. Flag Identify Network Deliver FH (FIND FH^®^), a machine learning phenotype-based approach developed by the FH Foundation, has a high precision (positive predictive value) for recognizing individuals with FH such that when flagged, 8 out of 10 times an individual, after evaluation by a clinician, is confirmed to have FH [10,18]. Genotype-based approaches, using whole exome sequencing in at-risk populations, are emerging as a method to identify individuals with FH who have a very high positive predictive value, but cost may be a factor [3]. While these phenotype- and genotype-based approaches show promise in identifying individuals with FH, systematic uptake has been slow [9,10,19,20,21]. System-level barriers to uptake include fragmented healthcare systems, lack of available data necessary for these approaches to work, and lack of interoperability among healthcare systems (i.e., poor communication between healthcare systems) [22]. 

Cascade testing, defined as the systematic screening of at-risk relatives, can also identify affected individuals with FH [23]. The historic method to improve family communication related to cascade testing is direct contact, whereby medical professionals obtain authorization from the index patient to share their protected health information with at-risk relatives. Successful direct contact programs have utilized a centralized approach for contacting at-risk relatives [23,24]. A novel new method is using chatbots, software applications that simulate conversation through text, developed for other health conditions. Chatbots have been used successfully to improve medication adherence and change health behaviors in breast cancer and mental health settings [25,26]. Chatbots have the potential to facilitate communication of information about FH on behalf of the index patients with FH and their at-risk relatives [11]. 

To improve uptake and success, we explored the implementation outcomes of acceptability, appropriateness, and feasibility of the distinct and complementary methods of identifying individuals with FH: automated screening approaches and innovative family communication methods. By acknowledging and addressing challenges to implementation prior to deployment, we can develop programs with the highest likelihood for success [27].

## 2. Materials and Methods

A protocol of the IMPACT-FH study has been published previously [28]. A summary of the methods relevant to this analysis are included here. This study was approved by the Geisinger Institutional Review Board. Stakeholders provided verbal consent by agreeing to, and subsequently participating in, focus groups. 

### 2.1. Study Population and Recruitment 

Individuals with FH and clinicians were invited by phone or e-mail to participate in focus groups exploring implementation outcomes prior to program deployment (Table 1).

Individuals with FH were recruited using convenience sampling from: (1) the FH Foundation Advocates for Awareness Training and (2) Geisinger patients with an FH diagnosis. Individuals who participated in the FH Foundation Advocates for Awareness Training gathered in Arlington, VA and had a clinical and/or genetic diagnosis of FH. Individuals who attended the Advocates for Awareness Training had been previously exposed to the idea of an automated screening approach, specifically the FH Foundation’s FIND FH approach, as part of the training. Individuals from Geisinger had a clinical diagnosis of FH on their problem list or a genetic diagnosis of FH from the MyCode Community Health Initiative^®^, a population genomic screening program linking blood, serum, and DNA samples to EHR data at Geisinger [29]. 

Clinicians were recruited using role-based and snowball sampling methods from three populations: (1) attendees of the 2020 National Lipid Association Spring Clinical Lipid Update; (2) Geisinger, an integrated healthcare delivery system in Pennsylvania; and (3) a primary care practice associated with a hospital in a small New England city. Clinician stakeholders included physicians, nurse practitioners, physician assistants, and pharmacists with expertise in cardiology, lipidology, endocrinology, primary care, and genetics. Attendees of the National Lipid Association meeting are national lipid experts and may have been previously exposed to automated screening approaches, such as FIND FH. 

### 2.2. Focus Group Procedures

**Figure 1:**
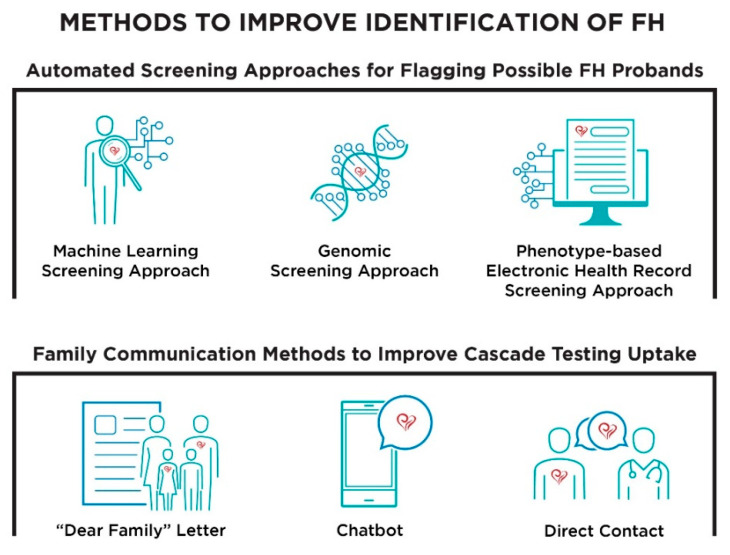
Visualization of the initiatives presented to stakeholders during focus groups.

**Figure 2:**
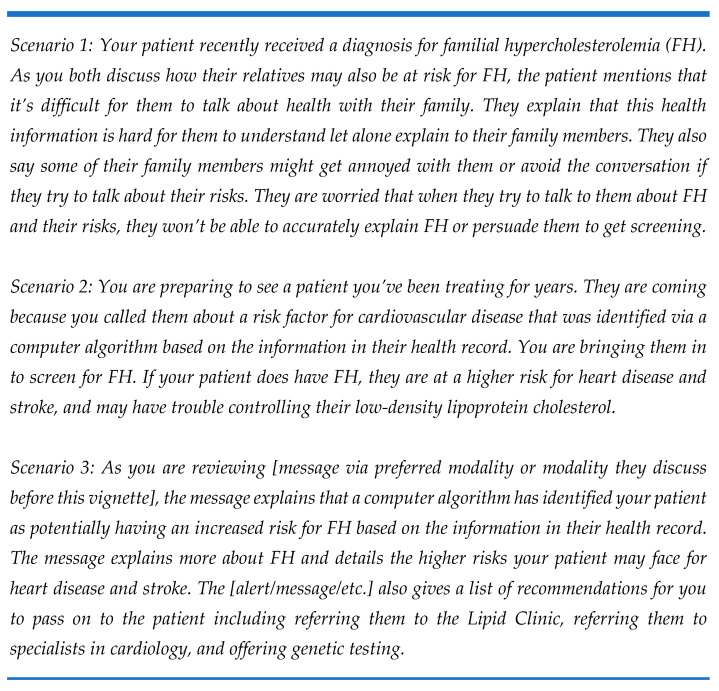
Excerpt of the scenarios presented to stakeholders during focus groups.

The Conceptual Model of Implementation Research [30,31] and previous literature about FH identification and cascade testing informed the development of the focus group guide and subsequent data analysis. The guide was structured to stimulate discussion about three implementation outcomes—acceptability, appropriateness, and feasibility—in relation to automated screening approaches and family communication methods to improve cascade testing (Material S1. Focus group guides). The family communication methods explored were a “Dear Family” letter, chatbot, and direct contact. Prior to the discussion of each initiative, the automated screening approach, chatbot, and direct contact were defined (Figure 1) and scenarios describing these were presented to the focus group participants (Figure 2). 

In-person focus groups were conducted by experienced qualitative researchers (L.K.J., A.K.R., and G.C.S.) and were approximately 1–1.5 h in duration. Due to the COVID-19 global pandemic, two of the planned in-person focus groups were held virtually, utilizing the Zoom^®^ platform. Only one stakeholder experienced connection or speech recording issues; to resolve this, selected text from the transcript was sent to the stakeholder for review. Each stakeholder was offered a $25 gift card for their participation. Each focus group was audio recorded with acknowledgement and verbal consent from stakeholders. Demographic surveys were collected from each stakeholder (Material S2 and S3. Demographic surveys). 

### 2.3. Data Analysis

**Figure 3:**
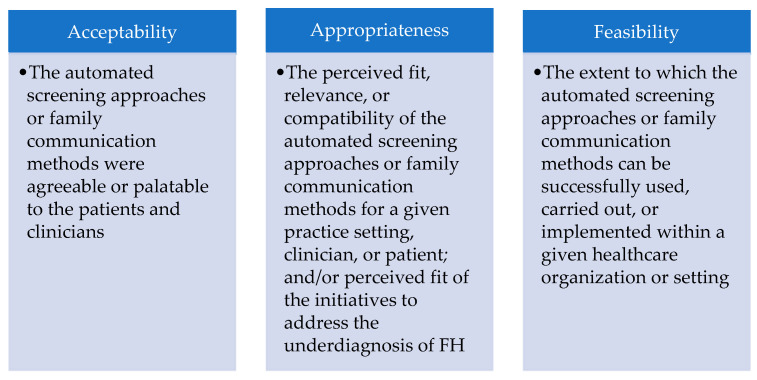
Study-specific definitions of the implementation outcomes: acceptability, appropriateness, and feasibility.

Utilizing a framework analysis [32], we reviewed focus group transcripts for discussion of the implementation outcomes acceptability, appropriateness, and feasibility (Figure 3).

**Figure 4:**
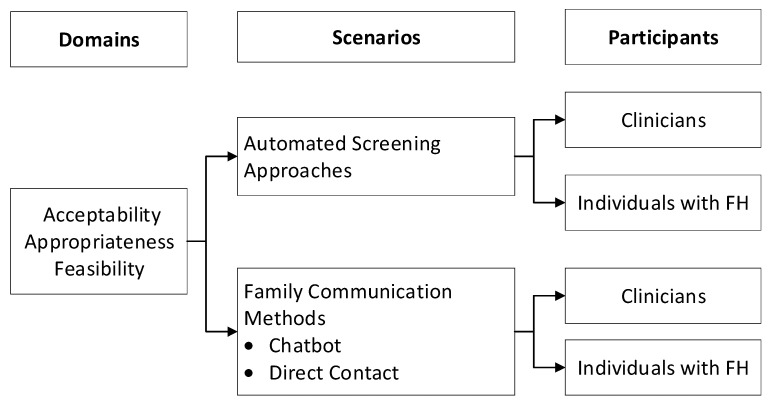
Analytic framework for reviewing domains by scenario and participants.

Audio-recordings from the focus groups were transcribed using a hospital transcription service and de-identified, checked for accuracy, and analyzed by the study team. A codebook was developed by study staff (L.K.J., A.B., and N.W.) using a deductive and inductive approach [33]. The deductive approach coded for the three implementation outcomes of interest (acceptability, appropriateness, and feasibility) and each initiative (automated screening approach, chatbot, and direct contact). The inductive approach captured themes expressed by participants related to the implementation outcomes of interest for each initiative. Our analytic framework is available in Figure 4. Four study team members (L.K.J., A.B., N.W. and M.G.) independently coded each transcript with discrepancies resolved by consensus with the rest of the coding team (A.K.R., A.C.S., and C.D.A.). After the four planned focus groups were conducted, thematic saturation was believed to be achieved, but missing representation from community-based clinicians lead to conducting a fifth focus group that confirmed that the presence of thematic saturation had been met [34]. Atlas.ti software version 8.4.15.0 was used to facilitate analysis and to compare themes across groups. 

## 3. Results

### 3.1. Demographics

A total of 22 individuals with FH and 20 clinicians participated in focus groups (Table 2). Patient participants had a clinical or genetic diagnosis of FH and were asked to hypothesize whether the automated screening approaches and novel family communication methods could be useful to identify patients who require a diagnostic evaluation for FH. 

### 3.2. Acceptability of Automated Screening Approaches and Family Communication Methods

#### 3.2.1. General Acceptability

All stakeholders reported general acceptance of both phenotype and genotype automated screening approaches to flag individuals with potential FH using health-related data and the novel family communication methods (Table 3). All stakeholders were concerned about, in the medical and patient communities, a general lack of awareness and knowledge of FH as well as a failure to differentiate FH as a distinct condition from other causes of hypercholesterolemia and, thus, requiring different medical care. They cited the overall low level of diagnosis of FH, and late diagnosis, as a significant reason for needing these types of initiatives. Individuals with FH found it acceptable to learn about FH from either of these proposed initiatives. Individuals with FH felt that repetition by various clinicians over multiple contact points would ensure that FH is acknowledged as a significant and concrete health threat that needs addressed. Clinicians felt these initiatives would help them reach an FH diagnosis earlier for their patients and at-risk relatives. 

#### 3.2.2. Acceptability Specific to Automated Screening Approaches

All stakeholders reported that earlier screening based on available health data would lead to earlier diagnosis and treatment of FH (Table 3). Individuals with FH found it acceptable to be “flagged” as potentially having FH because of an automated screening approach. These individuals wanted to be notified simultaneously with their clinician. Individuals with FH felt that notification would allow them time to research FH prior to future health encounters. Individuals with FH felt that then they would be able to come to prepared with questions for their clinician. Additionally, individuals with FH felt simultaneous notification would ensure that an FH evaluation would occur. Clinicians found it acceptable to implement the automated screening approaches, including simultaneous notification directly to individuals of potential risk for FH, and were also comfortable with other healthcare clinicians or staff reaching out to notify the clinicians’ patients of these results, as long as that initial outreach was done in their name, to maintain continuity of their relationship with the patient.

#### 3.2.3. Acceptability Specific to Family Communication Methods

All stakeholders reported that the novel family communication methods would streamline and simplify the pathway for individuals with FH to notify their relatives of their own FH diagnosis and the implications that this diagnosis might have for their relative while also managing delicate family dynamics (Table 3). Individuals with FH expressed how important it was to have flexible methods available to help them tailor and communicate their FH diagnosis with at-risk relatives. Individuals with FH liked the chatbot technology because it provided a digital platform for relatives to receive accurate medical information about FH, while providing critical information regarding the important next step of cascade testing. Individuals with FH mentioned that it was acceptable for healthcare teams or a centralized resource with national reach, such as the FH Foundation, to help relay the risks of FH and the need for screening to relatives (direct contact); however, individuals with FH wanted the opportunity to tell their relatives about their diagnosis first. Clinicians found it acceptable to reach out to patients‘ relatives directly to explain the diagnosis, host family meetings, or speak directly to a relative’s clinician. Clinicians expressed their understanding that it is acceptable to reach out directly to relatives (i.e., direct contact) from a privacy standpoint, as long as they were given permission by the index patient with FH to do so. However, clinicians did discuss concerns related to family dynamics that could impact the use of novel family communication methods, specifically lack of communication between some family members.

### 3.3. Appropriateness of Automated Screening Approaches and Family Communication Methods

#### 3.3.1. General Appropriateness

All stakeholders reported that the automated screening approaches and family communication methods would be appropriate ways to improve identification of individuals with FH (Table 4). Individuals with FH felt both approaches were suitable because it prompted either themselves or an at-risk relative to be screened with a family history risk assessment and a test (lipid panel and/or genetic testing for FH), which are normal clinical procedures, and to then discuss results with their clinician. Clinicians felt this was suitable because the initial testing process to diagnose FH requires non-invasive procedures. All stakeholders felt these initiatives were fitting because both approaches not only helped to diagnose an individual with FH earlier, but could also help promote family screening for FH.

#### 3.3.2. Appropriateness Specific to Automated Screening Approaches

All stakeholders expressed that automated screening approaches which utilize data available in a person’s health record were appropriate (Table 4). Individuals with FH found these automated screening approaches to be suitable because they felt automated approaches may have helped them be diagnosed with FH earlier and would have also enabled them to discuss FH with their relatives earlier. Clinicians found the automated screening approaches to be appropriate because such automated approaches would help them recognize a potential FH diagnosis they otherwise might have missed and would prompt the opportunity to also focus on diagnosis of other family members. Clinicians felt automated flags would be fitting in their practices because diagnosing FH earlier would help prevent early heart disease.

#### 3.3.3. Appropriateness Specific to Family Communication Methods

All stakeholders expressed the appropriateness of the novel family communication methods to improve discussions between themselves and relatives about the risks of FH (Table 4). Individuals with FH felt it was more suitable for them to try to reach out to their relatives first about the FH diagnosis, but saw the importance of also having healthcare teams or a centralized resource with national reach (i.e., FH Foundation) relay specific medical information and recommend next steps to their relatives. Individuals with FH discussed how the chatbot technology would be a more appropriate tool than direct contact for some relatives, especially relatives who were more technologically savvy. Clinicians noted that direct contact was appropriate because it allowed them to directly reach out to at-risk relatives with whom their patients asked them to discuss the relevance and importance of knowing about the FH diagnosis.

### 3.4. Feasibility of Automated Screening Approaches and Family Communication Methods

#### 3.4.1. General Feasibility

Overall, individuals with FH and clinicians thought the presented automated screening approaches and novel family communication methods could be feasibly implemented in current practice (Table 5). Both groups discussed ongoing initiatives in their own health systems that gave them confidence that these FH initiatives would be feasible to implement. Specifically, at one focus group, individuals with FH mentioned a genomic screening program that returned results using a similar process (i.e., MyCode Community Health Initiative). Individuals with FH also mentioned other healthcare apps (i.e., a menstrual cycle tracking app) they found useful for tracking health information. Clinicians mentioned pneumonia protocols, colon cancer and breast cancer screenings, and insurance company care gap lists as being similar to the FH initiatives. However, for these initiatives to be successful, both clinicians and individuals with FH explained there must be a plan to provide clinician training and education about FH in general (e.g., diagnostic criteria, treatment); as well as training on how these initiatives would be deployed in their local healthcare setting.

#### 3.4.2. Feasibility Specific to Automated Screening Approaches

All stakeholders reported that using an automated screening approach to identify individuals who require a diagnostic evaluation for FH would be practical (Table 5). One patient and one clinician focus group had prior exposure to the FH Foundation’s FIND FH^®^ approach and noted how the fact that this approach is currently being utilized in practice is one justification for its feasibility. Individuals with FH noted that it was important to receive a flag notice directly, rather than waiting for their next appointment, and also said it was reasonable to send these notifications in a variety of ways (e.g., mail, e-mail, telephone call) as no single method will work for everyone. Individuals with FH also described that it was important to draft the content of the notification in a way that would prompt the receiver to reach out to their clinician with some urgency, but also not be too alarming. Even though clinicians wanted the message about being flagged by the automated screening approach to come from them, they felt this would only be feasible if they had assistance from dedicated staff (e.g., extenders, medical assistants, nurses, nurse navigators, pharmacists, genetic counselors, other dedicated specialists) to help with the process. These staff members would make returning these flags more practical since they could also receive the notifications, notify the patients, and then coordinate next steps. These staff could order necessary diagnostic testing, provide continued patient communications, and potentially treatment, as well as provide external resources (i.e., FH Foundation). Clinicians noted that it would be important to have additional educational resources on FH available specifically for them. Such resources would better prepare them for a discussion with their patient and his/her at-risk relatives. Suggested examples included educational presentations at individual clinic sites, questions on state licensure exams, continuing education credits, podcasts, and social media.

#### 3.4.3. Feasibility Specific to Family Communication Methods

All stakeholders reported that having access to multiple communication options and the involvement of several healthcare team members to help facilitate discussion about FH with their relatives were practical (Table 5). Individuals with FH further noted the feasibility of programs to facilitate family communication by comparing such programs to tools provided by ancestry.com that helped them connect with extended family members. While individuals with FH wanted the ability to partner with a clinician when speaking with at-risk relatives, they felt it important to have a preliminary call/contact with their relatives to provide context for the conversation with a clinician. Some individuals with FH noted the chatbot would be an option for some relatives, especially if it was integrated into the health portal. Individuals with FH noted a variety of healthcare clinicians who could feasibly be their partner in directly reaching out to family members, including social workers, pharmacists, and genetic counselors. They also noted that it could be feasible for a patient advocacy organization, such as the FH Foundation, to assist in family outreach. Clinicians explained that direct contact of relatives would be more feasible if performed by staff outside of primary care (i.e., it was mentioned by primary care providers in our focus groups that even extenders in primary care such as nurse practitioners and physician assistants need their own extenders). These outside staff could be care managers or other supportive staff that have direct connections to patients for other reasons. Having the ability to refer to this type of dedicated staff member made the concept of partnering with patients in communicating directly with relatives feasible by alleviating a burden on the already busy clinician.

### 3.5. Perceived Obstacles to Implementation of Automated Screening Approaches and Family Communication Methods

All stakeholders noted some perceived obstacles to the implementation of automated screening approaches and novel family communication methods. No one obstacle was described as inhibiting the initiatives from being implemented into clinical practice; but addressing these obstacles would increase the likelihood of success. All stakeholders described the lack of awareness of FH as potentially limiting implementation of these initiatives due to health system and clinicians’ lack of understanding of the need to differentiate FH due to its high risk for premature cardiovascular disease. Individuals with FH shared concerns for themselves and for relatives about receiving an FH diagnosis, as they thought this might put them at risk of being denied health and/or life insurance. A failure to address these concerns could result in a decrease in uptake of these initiatives; however, it could be explained upfront that there are laws in place that protect individuals against genetic discrimination regarding health insurance specifically. However, explanations and education could be included upfront that while laws are in place to protect against genetic discrimination in health insurance, existing medical information such as abnormal lipid values and/or family history may be used for life insurance denials.

#### 3.5.1. Perceived Obstacles Specific to Automated Screening Approaches

All stakeholders highlighted the limited number of lipid specialists available to see identified individuals who require further diagnostic evaluation. Some individuals with FH also voiced specific questions related to privacy and permissions for access to the information used for the automated screening approaches, while others understood that this information is routinely available in the EHR and found this acceptable. Individuals with FH raised concerns about whether the results from automated screening approaches would be documented as a diagnosis within their health record without their knowledge, rather than a notification that further evaluation was needed. Clinicians discussed how fragmented healthcare systems result in incomplete patient records that could decrease the feasibility of an automated screening approach to identify individuals with potential FH because of insufficient or missing data. Clinicians also expressed concern that alerting them of flagged patients through the EHR may not be suitable because of significant “alert fatigue” within normal clinical practice. Clinicians were also concerned that missing these alerts would lead to delays in care and perhaps methods (e.g., InBasket messages, order sets) other than EHR alerts could be used to communicate flags to clinicians. Individuals with FH and clinicians questioned the availability of time and reimbursement for clinicians to return results from the automated screening approaches and to assist with the family communication among relatives. Clinicians felt this could be overcome utilizing dedicated staff and/or healthcare worker extenders in their practice.

#### 3.5.2. Perceived Obstacles Specific to Family Communication Methods

Both groups of stakeholders discussed privacy and confidentiality concerns related to the sharing of patients’ information with relatives that would make the family communication methods unacceptable if not addressed. Some of these concerns relate to a misunderstanding of what is allowed under the Health Insurance Portability and Accountability Act (HIPAA); such as, not understanding that clinicians can directly contact at-risk relatives to share information about the proband with FH, with permission from the proband to share their diagnosis. Clinicians noted, specifically regarding direct contact, that it may not be appropriate for them to reach out to relatives due to a concern for privacy and confidentiality and potential licensure regulations in each state that may prevent them from practicing medicine across state lines. Individuals with FH expressed concern regarding the possibility of breaches in their own privacy and confidentially of their personal information when using the family communication methods.

## 4. Discussion

We explored the potential acceptability, appropriateness, and feasibility of two initiatives—automated screening approaches and family communication methods—to help improve identification of FH in U.S. healthcare settings. All clinician and patient stakeholders indicated that automated screening approaches and family communication methods were acceptable, appropriate, and feasible to be utilized in real-world settings. Specific recommendations for implementation included (1) repetition by various clinicians over multiple contact points, (2) reporting results from the automated screening approaches to patients and clinicians at the same time, and (3) designing easy to use and clear family communication tools. The incorporation of physician extenders is likely needed to operationalize screening and family communication initiatives in busy practices. EHR notifications regarding the need for an FH evaluation should come in a way that is difficult for clinicians to ignore; for example, for those that use EPIC^®^ (Verona, WI), this would mean InBasket messages as opposed to Best Practice Alerts, with which clinicians have indicated fatigue. For successful implementation to occur, it will also be necessary to explain potential risks and address any misperceptions related to the ability of clinicians to share health and genetic information with at-risk family members, with authorization from the index patient with FH. Additionally, any misinformation related to the possibility of genetic discrimination after learning about an FH diagnosis would need to be addressed. For example, patients could be informed about the Genetic Information Nondiscrimination Act, which includes protection against health and employer discrimination based on genetic information, but does not provide protections related to life insurance.

Healthcare systems have pivoted to investing in automated methods to assist with many healthcare tasks, including identifying undiagnosed individuals for a variety of health conditions [9,10,17,18]. In particular, automated screening approaches to flag individuals who require a diagnostic evaluation for FH using machine learning, or other data mining approaches, have already been developed and tested worldwide [9,10,17,18]. Some believe that machine learning approaches may soon become diagnostic for FH [20] or will be able to predict the presence of a pathogenic genetic variant associated with FH [35]. While automated methods may help identify at-risk individuals, those methods that are confined to only notifying clinicians may not be the best choice. Physicians and other clinicians may miss alerts or have fatigue related to alerts, as noted above. In addition, we specifically heard from patient stakeholders that they want to be notified at the same time as their clinician so they can make sure appointments are scheduled and that they have the opportunity to learn about the condition prior to their next office visit. While these automated screening approaches have been tested via research for their ability to accurately identify individuals in need of evaluation, our study is the first, to our knowledge, to have investigated implementation science outcomes that will inform uptake of these automated screening approaches by individuals, clinicians, and healthcare systems.

Cascade testing facilitated by direct contact for genomic risk conditions has been successful in other countries, but direct contact has not been widely implemented in the U.S. and cascade testing uptake has also been slow [24,36,37] as it is met with substantial implementation barriers [38]. A recent pilot study of cascade testing for FH in primary care practices in the U.S. found an approximate 50% uptake of cascade testing by relatives, but noted limitations in data collection due to data privacy issues [39]. A recent systematic review of the literature found that cascade testing for FH is more effective when direct contact versus indirect contact is performed [23]. A recent evidence review and other literature have also found that direct contact by healthcare clinicians is preferred by some individuals and is also effective [40,41,42]. Further, a recent pilot program has shown potential feasibility of direct contact [37]. Despite being effective and being viewed as a valuable program by our participants, the implementation of direct contact has not yet been widely adopted. Stakeholders indicated that for direct contact to be acceptable, appropriate, and feasible, extenders dedicated to reaching out to relatives would need to be available and funded.

Chatbots may be highly useful in facilitating family communication as they address barriers to cascade testing identified at the individual, interpersonal, and environmental levels. These include suboptimal communication between patients with genetic conditions and their relatives; as well as geographic barriers to family communication, and in accessing genetic services, including genetic testing and counseling [38,43]. Chatbots can be used as a tool to improve communication between family members by providing scripted, factual information about FH and can also increase the accessibility of genetics education and cascade genetic testing.

Some healthcare systems have implemented programs for FH identification, where computer reminders invite eligible patients, identified by FH clinical diagnostic criteria, into primary care practices [44]. While the feasibility of these interventions has been measured [44], a recent systematic review of implementation strategies specific to improving statin uptake has found that implementation strategies have not been well defined nor systematically utilized and tested in practice [45]. The current study demonstrates the potential for and use of methodologies from implementation science for improving the identification of a common, high-risk genetic condition. Additional work to identify implementation strategies to improve uptake of genomic initiatives is needed for clinical integration [46].

A strength of this study is the direct engagement of individuals with FH and clinician stakeholders who have a range of personal experiences with FH diagnosis. However, our participants are not representative of the general population, since individuals with FH who participated in our focus groups were largely female, white, well educated in general, and well educated on FH. Another limitation of this study is that the initiatives discussed in the focus groups have not yet been widely implemented, so we were only able to measure perceived acceptability, appropriateness, and feasibility of these initiatives, as well as potential obstacles to their implementation. Due to the COVID-19 pandemic, we had to switch some of our in-person focus groups to a virtual format. Although the study team does not believe this affected our results, it is possible that the virtual approach may have affected group dynamics and participation.

## 5. Conclusions

Our results indicate that automated screening approaches, chatbots, and direct contact for FH diagnosis and cascade testing are considered acceptable, appropriate, and feasible to implement by patient and clinician stakeholders. We have identified factors that may impact implementation of such initiatives and that will need to be addressed for successful implementation.

## Figures and Tables

**Table 1:** Participant.

Focus Group	Stakeholder	Sample Representation	Invited	Participated
1	Individuals with FH	FH Foundation Advocates	18	15
2	Individuals with FH	Healthcare system	66	7
3	Clinician	Clinical lipid specialists	28	9
4	Clinician	Healthcare system	203	5
5	Clinician	Primary care practice	7	6

**Table 2:** Demographics of individuals with FH and clinicians.

Demographics		Value
Individuals with FH		22
	Female, n (%)	19 (86)
	White, n (%)	19 (86)
	Age range, years, n (%)	
	28–34	6 (27)
	35–54	7 (32)
	55 or older	9 (41)
	Higher educational obtainment, n (%)	
	Some college	4 (18)
	College graduate	12 (55)
	Post-graduate training	6 (27)
	Health insurance status, yes, n (%)	22 (100)
Clinicians		20
	Type, n (%)	
	Physician	15 (75)
	Advanced care providers (nurse practitioners, physician assistants, and pharmacists)	5 (25)
	Female, n (%)	7 (35)
	Race, n (%) *	
	White	14 (74)
	Asian	5 (26)
	Age range, years, n (%)	
	18–34	2 (10)
	35–54	6 (30)
	55 or older	12 (60)
	20 or more years of experience, n (%)	12 (60)
	Practice type, n (%)	
	Primary care	13 (65)
	Cardiology	4 (20)
	Others	3 (15)

* 1 declined to answer.

**Table 3:** Key points with exemplar quotes related to general acceptability and specific acceptability related to automated screening approaches and family communication methods.

Domain	Summary of Key Points	Exemplar Quotes
General	Knowledge and awareness of FH as a condition is low among community and clinicians Significance of FH as a specific condition is not well understood Risk factors and symptoms often overlooked by individuals and clinicians	*“[current standard] is one step better than asking the mortician to diagnose [FH]”* (Clinician, FG3) *“Ah, I don’t think [underdiagnosis of FH] is a lack of interest, but I think there is a lack of understanding of significance.* (Clinician, FG3) *“What we have missed the boat [on] is not diagnosing them with FH where we could impact their family members.”* (Clinician, FG5)
Automated approaches	Promotes earlier screening, flagging individuals who require a diagnostic evaluation based on available health data Notification about the need for screening to both individuals and clinicians Promotes individuals with FH to advocate for their own healthcare needs	*“Seems as though using this algorithm that will, it should help the doctors who aren’t specialists to call some attention to the possibility of FH. Because, when I think in my situation, yes, for years, I heard or every now and then that I had high cholesterol, but my primary care physician never knew I had it, was beating me up, saying I was eating the wrong things, only to find out later, after having an event, that the lipidologist said “Oh my gosh, you have FH.””* (Individual with FH, FG1) *“I think in hindsight, have been very helpful in our journey would have been if I, if somehow this algorithm flagged me, or, and then they automatically got flagged so that they got their screening at age 2 instead of this whacky way that we went about it and all of a sudden we had it. Um, and that it would, it would encourage pediatricians to follow the guideline, oh “potential FH risk, blood test now” versus this, like, weird thing.”* (Individual with FH, FG1) *“If there are tools to help me make that decision, that would be extremely welcomed…I can just treat it as elevated LDL and need to get it under 100 and so on.”* (Clinician, FG5) *“…I would like to be aware of it, but actually maybe, you know, it could be done and get the patients interested and willing to come in and talk about it without me having to even acknowledge it, it’s fine with me.”* (Clinician, FG5)
Family communication methods	A variety of methods are desirable to communicate with relatives Helps individuals and clinicians navigate family dynamics	*“I think [chatbots are] a great idea. Because you can choose to send it, you can choose to open it. It’s another tool.”* (Individual with FH, FG1) *“…[Family communication is] really I think individually based, but knowing as a patient, knowing the options and just knowing what choices you have may be the best option.”* (Individual with FH, FG2) *“I think most of us would be more than willing to have a family meeting if they wanna have people in. … If they wanted to, I certainly would. Whether it was by, you know, phone, or if they wanted to come in and bring family members with them.”* (Clinician, FG4)

**Table 4:** Key points with exemplar quotes related to general appropriateness and specific appropriateness related to automated screening approaches and family communication methods.

Domain	Summary of Key Points	Exemplar Quotes
General	Screening for FH was appropriate because it only requires a non-invasive procedure, which is routinely performed for individuals who present with high cholesterol anyway Promotes communication of risk and screening of at-risk relatives	*“They would realize that, and then the children, the relatives then should be recommended, and maybe that would be a part of this algorithm as well, is red flagging relatives that maybe haven’t gotten a–a lipid panel done, so when they go in for their next physical or so, to let the doctor know, ‘Hey this person has family risk of, uh, high cholesterol. Recommend a lipid panel to them.’”* (Individual with FH, FG1) *“I mean, I think you have a big advantage in that you’re basically asking people to get a blood test. Colonoscopies are, are a much harder thing to ask someone to do, a much bigger pitch. So, it…it’s something that most people have done, you know, yearly after a certain age and if they haven’t had it done for a couple years because they are younger and healthy, it’s generally not a big ask.”* (Clinician, FG4)
Automated approaches	Uses available health data to recommend evaluation for FH diagnosis which might have been missed via traditional screening approaches Enables individuals with FH to be identified earlier as well as relatives	*“I probably think it would make a lot of sense to use an algorithm because if it flagged it as FH specifically instead of just high cholesterol. If it was just high cholesterol, they would treat the patient as an individual. If it’s FH, they would treat the family.”* (Individual with FH, FG1) *“Build the algorithm into [electronic health record], so that when we are ready to mistakenly just click on dyslipidemia or elevated cholesterol, it will guide us to the correct… ‘Have you considered?’ and it will pop up. ‘Have you considered familial hypercholesterolemia?’”* (Clinician, FG5)
Family communication methods	Individuals with FH felt it was appropriate for them to first reach out to their relatives Chatbot technology would be appropriate method to contact some relatives Direct contact approaches were suitable to both individuals with FH and clinicians to discuss risk of FH with relatives	*“I would like the opportunity to speak with my family first and then if I find some reluctancy from my family, then getting a, um, a doctor that is involved, but I would prefer it to be a lipidologist, because I feel–or a cardiologist–some type of specialty or genetic counselor, to put–I think that puts the fire under somebody’s butt.”* (Individual with FH, FG1) *“… I have a teenage sister, and she would not talk to somebody on the phone. She would absolutely text a bot over talking to somebody.”* (Individual with FH, FG2) *“If it’s just one or two family members then I would offer the patient that I would call them and explain that this is what they have and this is what they should do, but if it’s a big family then, then I would…if they wanna come in and have a family meeting or something then I would be willing to do that. But I’m not going to call, like, 20 different family members and explain them individually that this is something you’ve gotta do and this is something you need to be tested for.”* (Clinician, FG4)

**Table 5:** Key points with exemplar quotes related to general feasibility and specific feasibility related to automated screening approaches and family communication methods.

Domain	Summary of Key Points	Exemplar Quotes
General	Feasibility because of similarity to other initiatives To be successful, clinical staff training and education must occur	*“When I’ve had an unusual breast exam and colon... I got a letter, a phone call, and a letter. But they weren’t from my doctor. They were from where I went, clinic or whatever, to get the exam. And they said something very professional, ‘Your, uh, reading was abnormal. We’d like for you to schedule an appointment to come back in.’”* (Individual with FH, FG1) *“…we’re already doing [automated approaches] as [another participant] said with…with colon cancer.”* (Clinician, FG4) *“This sounds like an opportunity to educate the physicians … So, I guess this is an opportunity you can teach us and I guess help us reach other people that may not have access to or may not be involved with a physician.”* (Clinician, FG5)
Automated approaches	Notification in a variety of ways (mail, e-mail, telephone) Need for dedicated staff/team to manage notification and coordination of automated screening approach results	*“So, I say do it all. Send it by mail. Give them a call. Send an email because it’s just, each person receives it differently.”* (Individual with FH, FG2) *“We really should be approaching this from a team-based approach instead of trying to funnel every piece of information and every decision through the primary care physician. I mean, if you want to do that, you can go hang your shingle up down the street. But the whole idea of having a team-based approach is that we are improving the quality of care. At the same time, we’re letting our providers get home in time to have dinner with their family.”* (Clinician, FG4)
Family communication methods	Chatbot is feasible to use, especially if integrated into their patient portal Direct contact is feasible to do but would require permission from the patient and information on their relatives Various types of clinicians could partner with patients in communicating with their relatives	*“Linking [the chatbot] to your [patient portal] I think would be…because that’s how I communicate with my doctors is through [the patient portal], so having that information in there I think would be beneficial.”* (Individual with FH, FG2) *“So, with the permission of the family member or the patient, to speak to others I mean, I don’t have any hesitation trying to, you know, convince them to go and get tested. So, um, if it was, you know, like the index patient, my patient had hesitation about giving me information or sharing anything with anyone else, that might be a different story.”* (Clinician, FG5)

## Data Availability

The data presented in this study are available in the published article or supplemental files.

## Supplementary Materials

The following are available online at https://www.mdpi.com/article/10.3390/jpm11060587/s1, Material S1. Focus group guides, Material S2 and S3. Demographic surveys.
