# Supplementary material for: Acceptability, Appropriateness, and Feasibility of Automated Screening Approaches and Family Communication Methods for Identification of Familial Hypercholesterolemia: Stakeholder Engagement Results from the IMPACT-FH Study
Source: J Pers Med. 2021 Jun 21;11(6):587. doi: 10.3390/jpm11060587 (PMC8234213; doi:10.3390/jpm11060587)
Supplement: Supplementary file 1 [file jpm-11-00587-s001.zip › jpm-1239847-supplementary.pdf]

Supplemental Materials

S1. Focus group guide……………………………………………………………………….Pages 2-6

S2. Individual with FH Demographic Sheet…………………………………………………Pages 7-8

S3. Clinician Demographic Sheet.......………………………………………………………Pages 9-10

FOCUS GROUP GUIDE

[Focus group*: Welcome participants and have everyone fill out name tent and grab snacks/dinner as they* arrive. Begin to fill out demographic sheet as everyone gets settled.]

[Interview*: Participants will be read the demographic sheet at the end of the interview.*]

Welcome

Hello, my name is [Name] from Geisinger.

Thank you for agreeing to take part in this study. As a reminder, your participation in this study is voluntary. If you decide not to be in this study, it will not affect your [employment/access to health care]. As you recall, we expect this [focus group] to take approximately [2 hours].

Consent/Ground Rules

The purpose of this [focus group] is to discuss communication with patients and at-risk family members about familial hypercholesterolemia, known as FH. Everything you tell us will be kept confidential among the research team and nothing in our report will identify you individually. We are looking at improving our communication with patients and family members. Your feedback will be very helpful as we combine your comments with information from other patients and providers both at Geisinger and nationally.

We would like to go over some ground rules for the focus group before we begin.

Ground rules:

- *All opinions matter; please try not to talk over each other.*
- *We may call on individuals to make sure everyone has a chance to speak.*
- *We may cut off a conversation to move on, if necessary.*
- *Be respectful - Disagree with ideas, not with people.*
- *Vegas rules - Do not discuss other participants' information or stories outside of the group.*
- *We are recording this discussion, but no names will be connected to what you say.*

This [focus group] will be recorded to create a transcript for analysis, but your identifying information will not be included on the transcript. You may choose to not answer any question and mayleave the [focus group] at any time.

May I please continue with the recording?

FOCUS GROUP ONLY: Roundtable Introduction of Participants

*Let's go around the table. Please introduce yourself using your first name and tell the group what*motivated you to participate today

PART I: ACCEPTABILITY AND FEASIBILITY OF EHR IDENTIFICATION AND FAMILY COMMUNICATION

A. PATIENTS

Education

[Describes and defines FH]

Automated screening approaches

Vignette**:**

[Slide that describes how a new tool - an algorithm to find FH - works. Explanation of how this algorithm analyzes the individual's EHR. Then the algorithm can identify who in a healthcare system is at higher risk for FH. In the past, this information has been shared with the individual's provider. The provider is then encouraged to share this with their patient and consider follow up testing to figure out whether the patienthas FH].

Imagine you receive a call from your physician asking you to come in for an appointment to discuss your high cholesterol because they have learned some new information. When you get to your appointment, the physician says there's a new tool that can help identify individuals at higher risk for heart disease. Your provider explains that with this new tool a computer analyzed your health record and based on the information in your health record – the system has identified you as potentially having a higher risk for heart disease than most other people with high cholesterol. Your provider recommends that you do further testing to see if you have FH. When test results are returned, your provider diagnoses you with FH.

Question: *What do you think about this?*

Probes:

1. *What about this would work for you?*
2. *What might concern you about this?*
3. *What would you do?*
4. *What else is going through your head?*
5. *What would you do next after hearing this information from your provider?*

Question: *How would you feel about learning your possible FH diagnosis through this process?*

Probes:
1. *Thoughts about the algorithm prompting the process for your doctor?*
2. *…feelings about it coming out of the blue?*

Question: *How could this be done better?*

***Probes**:*

1. *From your experience being diagnosed with FH, what could we do better?*

2. *What's the best way to tell individuals identified by an algorithm about their possible risk for FH?*
    a. *How do you think someone at risk should be notified?*
    b. *What should we say when explaining this to an individual?*
3. *When might a tool like this have been most useful for you? Family members?*

PART I: ACCEPTABILITY AND FEASIBILITY OF EHR IDENTIFICATION AND FAMILY COMM. (Cont.)

Family Communication

Vignette**:**

*Your doctor tells you it's very likely that others in your family also have this condition. Family members like*your siblings, children, parents, and cousins could have FH as well. Your doctor tells you that your family members also need to be screened for FH.

Question: *Your doctor asks, "What do you think would be the best way to alert your family members to* their risks, so they get screened for FH?"

Probes:

1. *Would you be willing to share this information with family members?*
2. *How do you think your family members would react to receiving this information?*
3. *How would you share this information?*

    Probes if no response*: (give additional "what about …" suggestions below AFTER they*answer)
    a. *Have a healthcare provider contact family member? Chatbot? (describe chatbot) Personally give the family member a letter?*
    b. *Would you use different communication methods or say different things to different family members?*
4. *How could we help you share this information?*
    a. *How would you feel about a provider contacting your family members for you?*
        1. *How would you want this to work?*
        2. *How comfortable would you be giving a provider/healthcare system your relative's contact information?*
    b. *What type of healthcare professional – nurse, doctor, genetic counselor, etc. – would you prefer to contact your family members?*
    c. *How would you feel about a third-party organization like the FH Foundation contacting your family members?*
5. *What information would you expect your provider to share?*
    a. *What information would you feel uncomfortable with your provider sharing?*
6. *Have you had any experience with receiving information about a health condition other than FH?*
    a. *How was that information communicated with you?*
    b. *What were your feelings about that method of communication?*

PART I: ACCEPTABILITY AND FEASIBILITY OF EHR IDENTIFICATION AND FAMILY COMM. (Cont.)

B. PROVIDERS

Educational

Slide

[Describes and defines FH.]

Family Communication

Vignette:

Imagine your patient recently received a diagnosis for familial hypercholesterolemia (FH). As you both discuss how their relatives may also be at risk for FH, the patient mentions that it's difficult for them to talk about health with their family. They explain that this health information is hard for them to understandlet alone explain to their family members. They also say some of their family members might get annoyedwith them or avoid the conversation if they try to talk about their risks. They are worried that when they try to talk to them about FH and their risks, they won't be able to accurately explain FH or persuade themto get screening.

Question: *They ask if you can help them tell their family in some way…*

Probes:

1. *What if they want YOU to contact the family member?*
   a. *How do you feel about that?*
   b. *What do you say (to the patient asking)?*
2. *What would make it difficult to contact family members?*
   a. *Relatives outside healthcare system? Billing/insurance?*

EHR

Vignette A:

[Slide that describes how a new tool - an algorithm to find FH - works. Explanation of how this algorithm analyzes the individual's EHR. Then the algorithm can identify who in a healthcare system is at higher risk for FH. In the past, this information has been shared with the individual's provider. The provider is then encouraged to share this with their patient and consider follow up testing to figure out whether the patienthas FH].

Imagine you are preparing to see a patient you've been treating for years. They are coming because you called them about a risk factor for cardiovascular disease that was identified via a computer algorithm based on the information in their health record. You are bringing them in to screen for FH. If your patient does have FH, they are at a much higher risk for heart disease and stroke, and may have trouble controlling their LDL cholesterol.

Question: *What do you think about this algorithm approach?*

Probes:

1. *What about this would work well for you?*
2. *What might concern you about this??*
3. *What would you do?*
4. *What else is going through your head?*
5. *How would you like to see a message prompting you that the patient is at risk for FH andneeds further screening?* (Note the preferred modality of the group.)

PART I: ACCEPTABILITY AND FEASIBILITY OF EHR IDENTIFICATION (Cont.)

Vignette B:

As you are reviewing [message via preferred modality or modality they discuss before this vignette], the message explains that a computer algorithm has identified your patient as potentially having an increasedrisk for familial hypercholesterolemia (FH) based on the information in their health record. The message explains more about FH and details the higher risks your patient may face for heart disease and stroke. The [alert/message/etc.] also gives a list of recommendations for you to pass on to the patient including referring them to the Lipid Clinic, referring them to specialists in cardiology, and offering genetic testing.

Question: *You begin to think about how you should handle this information and what to do next to guide*your patient's care…

Probes:

1. *What would you need to help you guide this patient?*

2. *How comfortable would you feel managing this patient?*

3. *What do you want the healthcare system to do to help you with these patients?*

Individual with FH Demographic Sheet

Study ID: ___________________

1. What is your sex?
   - ☐ Male
   - ☐ Female
   - ☐ Prefer not to answer

2. Which of the following best describes your age group?
   - ☐ 18 - 24
   - ☐ 25 - 34
   - ☐ 35 - 44
   - ☐ 45 - 54
   - ☐ 55 - 64
   - ☐ 65 or older
   - ☐ Prefer not to answer

3. What is the highest level of education that you have completed?
   - ☐ Grade school/junior high
   - ☐ Some high school
   - ☐ High school graduate
   - ☐ Trade/technical/vocational school
   - ☐ Some college
   - ☐ College graduate
   - ☐ Post graduate work or graduate degree
   - ☐ Don't know
   - ☐ Prefer not to answer

4. What type(s) of health insurance coverage do you have? (**Please select all that apply**)
   - ☐ I do not have health insurance
   - ☐ Private insurance (for example, Geisinger Health Plan or Blue Cross Blue Shield)
   - ☐ Medicaid
   - ☐ Medicare
   - ☐ Tricare/military
   - ☐ Don't know
   - ☐ Prefer not to answer

5. Do you consider yourself to be of Hispanic or Latino origin?
   - ☐ Yes
   - ☐ No
   - ☐ Don't know
   - ☐ Prefer not to answer

6. With which race do you identify yourself?
   - ☐ Black or African American
   - ☐ White
   - ☐ Asian
   - ☐ American Indian or Alaskan Native
   - ☐ Native Hawaiian or other Pacific Islander

- ☐ Other (Please specify): ____________________
- ☐ Prefer not to answer

7. Are you currently married or living with a partner?
    - ☐ Yes
    - ☐ No
    - ☐ Prefer not to answer

8. Are you currently working for pay?
    - ☐ Yes
    - ☐ No
    - ☐ Prefer not to answer

9. Please indicate <u>your total combined </u>yearly **household** income before taxes.
    - ☐ < $15,000
    - ☐ $15,000 - $30,000
    - ☐ $30,001 - $50,000
    - ☐ $50,001 - $75,000
    - ☐ $75,001 - $100,000
    - ☐ $100,001 - $150,000
    - ☐ $150,001 - $200,000
    - ☐ > $200,000
    - ☐ Don't know
    - ☐ Prefer not to answer

10. How often do you have problems learning about your medical condition because of difficulty understanding written information?
    - ☐ Always
    - ☐ Often
    - ☐ Sometimes
    - ☐ Occasionally
    - ☐ Never
    - ☐ Prefer not to answer

11. How often do you have someone help you read materials about health care (for example, the materials that you might receive if you are in the hospital or at your doctor's office)?
    - ☐ Always
    - ☐ Often
    - ☐ Sometimes
    - ☐ Occasionally
    - ☐ Never
    - ☐ Prefer not to answer

12. How confident are you filling out medical forms by yourself?
    - ☐ Extremely
    - ☐ Quite a bit
    - ☐ Somewhat
    - ☐ A little bit
    - ☐ Not at all
    - ☐ Prefer not to answer

Clinician Demographic Sheet

Study ID: ___________________

13. What is your sex?
    - ☐ Male
    - ☐ Female
    - ☐ Prefer not to answer

14. Which of the following best describes your age group?
    - ☐ 25 - 34
    - ☐ 35 - 44
    - ☐ 45 - 54
    - ☐ 54 - 64
    - ☐ 65 or older
    - ☐ Prefer not to answer

15. Do you consider yourself to be of Hispanic or Latino origin?
    - ☐ Yes
    - ☐ No
    - ☐ Don’t know
    - ☐ Prefer not to answer

16. With which race do you identify yourself?
    - ☐ Black or African American
    - ☐ White
    - ☐ Asian
    - ☐ American Indian or Alaskan Native
    - ☐ Native Hawaiian or other Pacific Islander
    - ☐ Other (Please specify): ___________________
    - ☐ Prefer not to answer

17. What is your area of work/practice?
    - ☐ Primary care
    - ☐ Cardiology
    - ☐ Endocrinology
    - ☐ Genetics
    - ☐ Pharmacy
    - ☐ Other (Please specify): ___________________

18. Please select the best option to describe your job:
    - ☐ Physician
    - ☐ Advanced Practitioner
    - ☐ Genetic Counselor
    - ☐ Pharmacist
    - ☐ Nutritionist

19. How many years have you been in practice (e.g. since residency)?
    - ☐ <5
    - ☐ 5-10

- ☐ 10-15
- ☐ 15-20
- ☐ >20

20. How many years have you been at your current institution?
    - ☐ <5
    - ☐ 5-10
    - ☐ 10-15
    - ☐ 15-20
    - ☐ >20

21. How many years have you been in your current position?
    - ☐ <5
    - ☐ 5-10
    - ☐ 10-15
    - ☐ 15-20
    - ☐ >20

22. How often do you see patients with an FH diagnosis?
    - ☐ I don’t see patients with an FH diagnosis
    - ☐ Less often than monthly
    - ☐ About once a month
    - ☐ A few times a month
    - ☐ About once a week
    - ☐ A few times a week
    - ☐ Daily
